# High diversity of small insectivorous mammals on Qinghai–Tibet Plateau and first description of karyotype for four endemics of China

**DOI:** 10.1038/s41598-021-03809-4

**Published:** 2021-12-30

**Authors:** Svetlana V. Pavlova, Vladimir S. Lebedev, Vasily D. Yakushov, Yongke Zhu, Yun Fang, Yue-Hua Sun, Boris I. Sheftel

**Affiliations:** 1 A.N. Severtsov Institute of Ecology and Evolution, Russian Academy of Sciences, 33 Leninsky pr., 119071 Moscow, Russia; 2 Zoological Museum, Moscow State University, Moscow, Russia; 3 Key Laboratory of Animal Ecology and Conservation Biology, Institute of Zoology, Chinese Academy of Science, Beijing, 100101 People’s Republic of China

**Keywords:** Cytogenetics, Taxonomy

## Abstract

Among seven species of the order Eulipotyphla (from southern Gansu and northern Sichuan Provinces, Central China) studied cytogenetically, karyotypes of one talpid species, *Uropsilus aff. soricipes* (2n = 36, NF*a* = 54), and three soricid species, *Chodsigoa hypsibia* (2n = 65, NF*a* = 66), *Sorex cansulus* (2n = 42, NF*a* = 64) and *Sorex thibetanus* (2n = 42, NF*a* = 60), are described cytogenetically for the first time. All four species are endemic to China with distribution ranges restricted to the Qinghai–Tibet Plateau and adjacent mountain ranges. The *Ch. hypsibia* karyotype consists of mostly acrocentric autosomes and one metacentric pair of autosomes; besides, a B chromosome was identified. No polymorphism was detected among karyotypes of other species, including shrews *Sorex bedfordiae* (2n = 26, NF*a* = 44), *Anourosorex squamipes* (2n = 48, NF*a* = 92) and *Crocidura suaveolens* (2n = 40, NF*a* = 44). The Chinese shrew mole *U. aff. soricipes* and three shrew species (*S. bedfordiae*, *Ch. hypsibia* and *A. squamipes*) represent autochthonous fauna of Central/Western China, whereas *S. thibetanus*, *S. cansulus* and *C. suaveolens* belong to phylogenetic groups occurring mostly to the north and west from China; therefore, they should be considered relatively recent colonisers. Thus, considering the relationships of the species within phylogenetic groups, our results on karyotypes are in good agreement with molecular genetic data.

## Introduction

The territory of China is characterised by high species diversity of insectivorous mammals (Eulipotyphla) including 13 genera of shrews (Soricomorpha), seven genera of moles (Talpomorpha) and six genera of hedgehogs and gymnures (Erinaceomorpha)^1–6^, some of which are relict representatives of highly divergent ancient lineages (e.g. *Uropsilus* and *Anourosorex*). At the same time, certain genera (e.g. *Chodsigoa*, *Episoriculus* and *Uropsilus*) have their centres of extensive radiation in southern and central China^2^; this observation explains high richness and endemism at the species level. In recent years, the rate of new species discovery increased substantially. According to seminal ‘A Guide to the Mammals of China’ (2008)^5^, Chinese fauna includes only 72 eulipotyphlan species (seven species of hedgehogs, 15 species of moles and 50 species of shrews), while ‘The Mammals of the World’ published in 2018 lists as many as 87 species (nine species of hedgehogs, 20 species of moles and 58 shrews)^2,3,6^, not including two more species of hedgehogs (*Mesechinus miodon* and *M. wangi*), which were recognised and described in a later revision^4^, and a recently reported new monotypic genus of scalopine moles (*Alpiscaptulus*)^1^.

Most of the newly described species have been discovered in China by molecular genetic methods, which continue to play an increasingly important role in the identification of cryptic species^7–9^. Nonetheless, variation in other characteristics that can be informative for taxonomic purposes is still studied insufficiently, including parameters of karyotypes. Currently, there are no published cytogenetic data on 40% of species of Chinese shrews (23 species) and moles (nine species), while all species of hedgehogs inhabiting China are already karyotyped^3,10–12^.

Chromosomal information on many autochthonous species has been unavailable, including endemics and sub-endemics of China (for example, members of genera *Sorex*, *Uropsilus*, *Nectogale*, *Chimarrogale* and *Euroscaptor*). For cytogenetically analysed species, often only routinely stained sets of chromosomes are reported (for example, for *Anourosorex squamipes*, *Uropsilus andersoni*, *Scaptonyx fusicaudus* and *Episoriculus sacratus*); this is certainly not enough to establish relationships between closely related species^13^. It should be emphasised that cytogenetic data have greatly contributed to the progress in the systematics^14–16^ of many groups of Eulipotyphla (e.g. *Sorex*). A high rate of chromosomal rearrangements can facilitate speciation via fixation of incompatible chromosomal variants in geographically isolated populations^17,18^. Considering that complex topography and climatic history of the eastern Qinghai–Tibet Plateau (QTP) have definitely promoted habitat fragmentation and population isolation, one may expect that at least in some groups, there would be high levels of chromosomal variation among otherwise cryptic lineages. Accordingly, the aim of our work was to present new cytogenetic data along with the first description of karyotypes of several insectivores from the eastern edge of the QTP, by focusing on Minshan. Some of these species are narrow-range local endemics (*Sorex cansulus*), while others belong to species complexes occurring throughout Hengduan and adjacent territories (*S. bedfordiae*, *Uropsilus* sp*.* and *A. squamipes*) or even across Eurasia (*Crocidura ex gr. suaveolens*).

## Results

**Figure 1:**
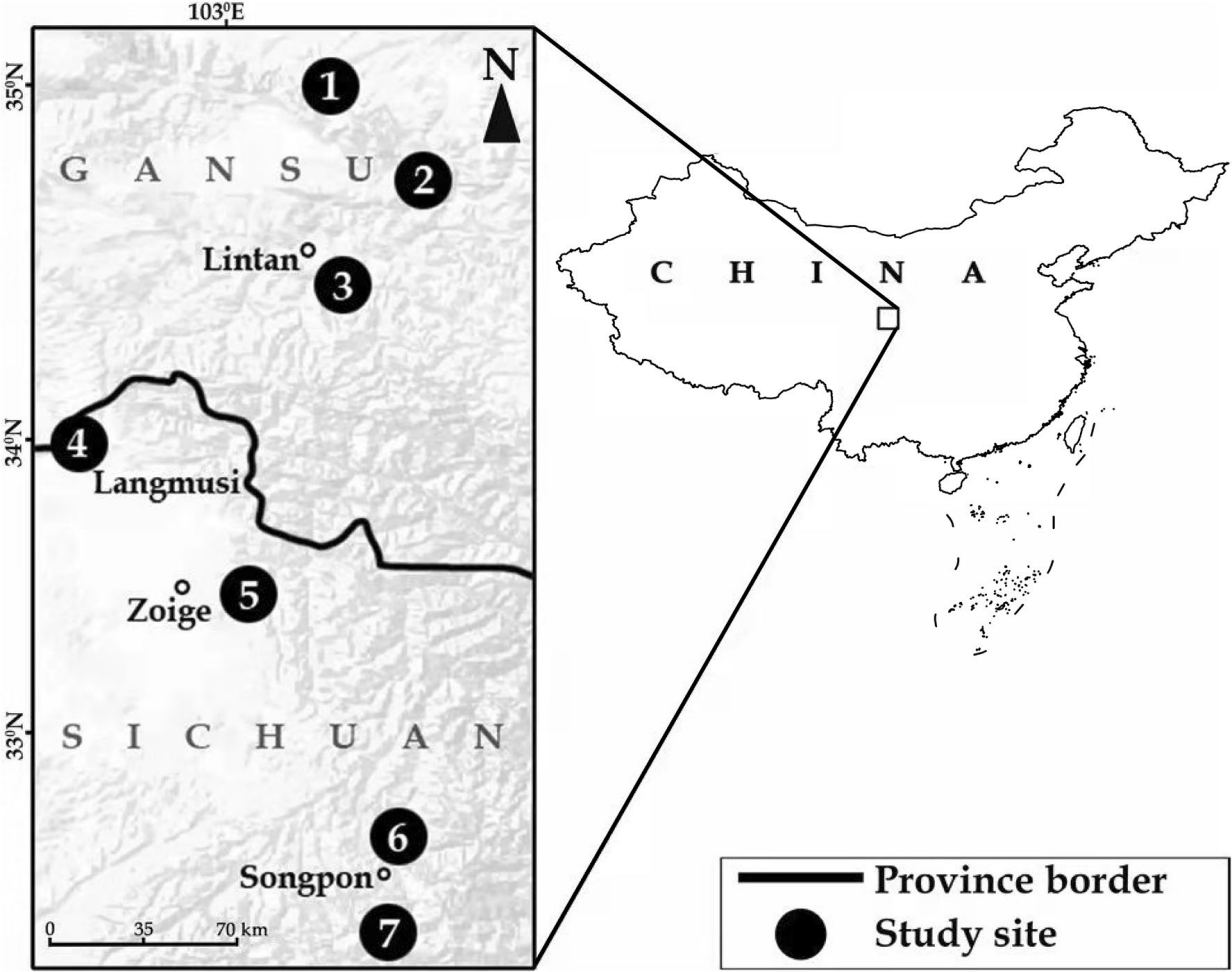
The map of the sampling sites of the karyotyped small mammals in China. (1) Taizishan National Nature Reserve, Hubei County, Gansu Province, 2500 m a.s.l., N35° 14′, E103° 25′; (2) Lianhuashan National Nature Reserve, Gansu Province, 3100 m a.s.l. N34° 55′, E 103° 44′; (3) Zhuoni County, Gansu Province, right bank of Tao River, 2600 m a.s.l., N34° 34′, E103° 27′; (4) Hongxing town (Langmusi), Songpan County, Sichuan Province, 3450 m a.s.l., N34° 04′, E102° 37′; (5) Banioucun, Zoige County, Sichuan Province, 3400 m a.s.l., N33° 35′, E103° 09′; (6) Chuanzhusi town, Songpan County, Sichuan Province, 3300 m a.s.l., N32° 45′, E103° 38′; (7) Xiaobaosi vil., Songpan County, Sichuan Province, 3000 m a.s.l., N32° 29′, E103° 35′. The map was made using QGIS (v.3.16) (QGIS Geographical Information System, https://qgis.org/ru/site/). A map layer was taken from China Standard Map Service (http://bzdt.ch.mnr.gov.cn/). The figure was created in Adobe Photoshop (v 21.2.0) (https://www.adobe.com/ru/products/photoshop.html) by V. D. Yakushov.

Seven insectivorous species belonging to five genera and two families, Talpidae and Soricidae, were cytogenetically studied. Karyotypes of four endemics of China (*Uropsilus aff. soricipes*, *Sorex thibetanus*, *S. cansulus* and *Chodsigoa hypsibia*) are described for the first time; as for the remaining species, new additional information is provided (Table 1, Fig. 1).

**Table 1:** A summary of karyotypes of the analysed species collected in China. Only a female individual of *Sorex thibetanus* was karyotyped, and therefore sex chromosomes could not be identified. N, sample size; 2n, diploid chromosomal number; NF*a*, fundamental number of autosome arms; M/SM, metacentric or submetacentric; ST, subtelocentric; A, acrocentric; B, B chromosome; X and Y, sex chromosome. Species karyotyped for the first time are highlighted in bold.

Species	N	2n	NF*a*	M/SM	ST	A	B	X	Y
Family Talpidae *** Uropsilus aff. soricipes***	1	36	54	2	8	7	–	M	dot A
Family Soricidae * Sorex bedfordiae*	9	26	44	8	2	2	–	A	A
*** Sorex thibetanus***	1	42	60	9	1	10	–	A	?
*** Sorex cansulus***	3	42	64	11	2	7	–	A	dot A
* Anourosorex squamipes*	2	48	92	23	0	0	–	M	ST
*** Chodsigoa hypsibia***	1	65	66	1	0	30	0/1	A	A
* Crocidura suaveolens*	1	40	44	2	1	16	–	ST	A

### Family Talpidae

**Figure 2:**
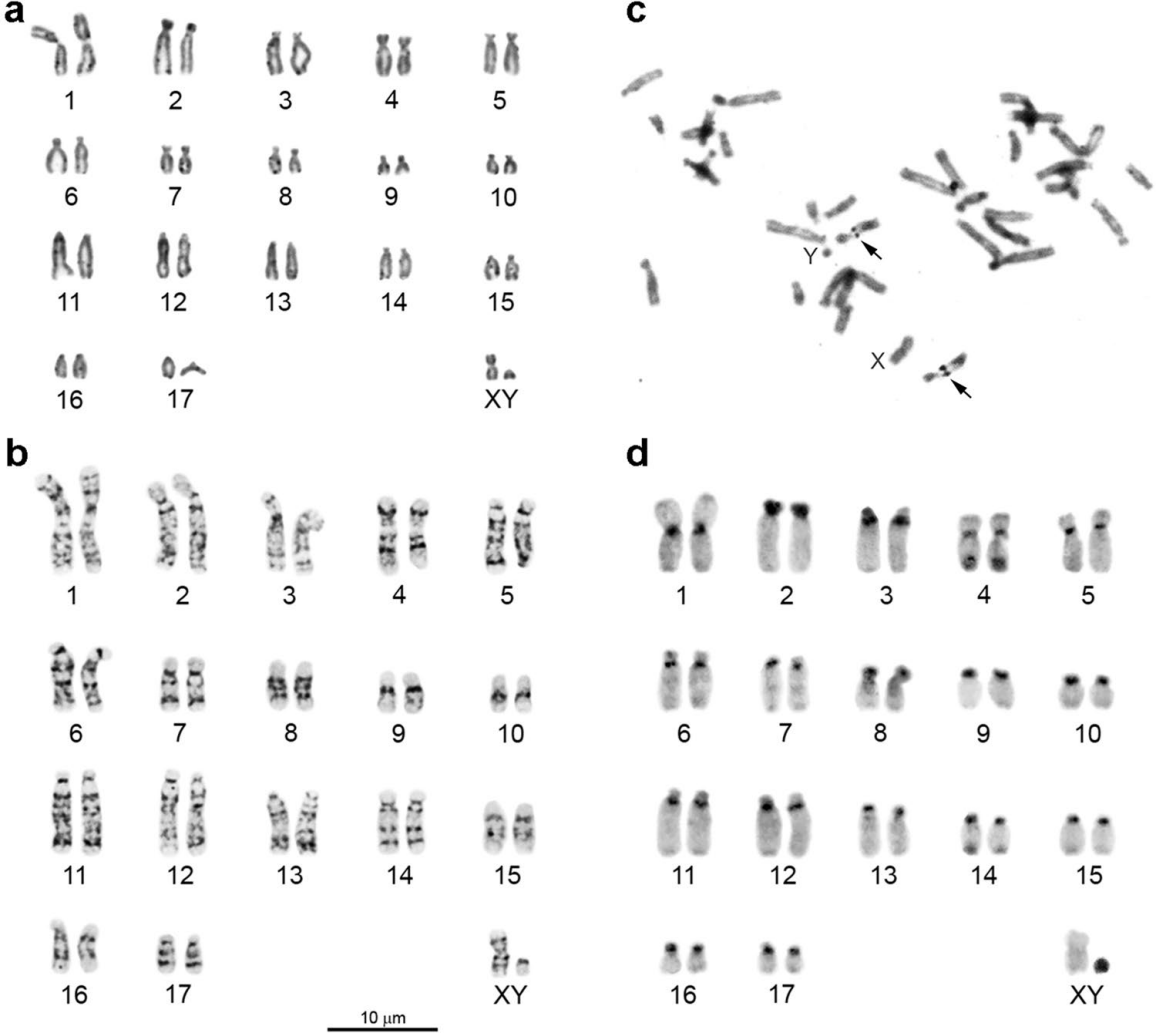
A male karyotype of *Uropsilus soricipes* from Sichuan Province (Zoige). (**a**) Conventional staining, (**b**) G-banding, (**c**) C-banding and (**d**) silver nitrate staining. Arrows indicate nucleolus organiser regions (NORs). 2n = 36, NF*a* = 54. XY: sex chromosomes.

One male individual of the Chinese shrew mole *U. aff. soricipes* Milne-Edwards, 1871, endemic to China (G18-213), was collected at site No. 3. The karyotype is presented for the first time: 2n = 36, NF*a* = 54 (Fig. 2a). The autosomal set consists of 10 bi-armed and seven single-armed chromosomes: one large metacentric pair (chromosome 1) and one mid-size submetacentric pair (chromosome 4), eight large-to-small subtelocentric pairs (chromosomes 2, 3 and 5–10) and seven large-to-small acrocentric pairs (chromosomes 11–17). After the G-banding pattern was assessed, the small metacentric and smallest acrocentric were identified as X and Y chromosomes, respectively (Table 1, Fig. 2b).

Silver-positive dots were tentatively registered close to centromeres in one mid-size subtelocentric pair, chromosome 4 (Fig. 2c).

C-positive heterochromatic blocks were found to be located in centromeric regions of all autosomes except for two subtelocentric pairs, chromosomes 2 and 8, which have fully heterochromatic short p-arms. Visible C-blocks were revealed at a terminal position of q-arms in two autosome pairs, chromosomes 4 and 14. Less darkly stained C-bands were visible at interstitial positions of pair number 8. The X chromosome is C-negative, whereas the Y chromosome stains positively throughout the entire chromosome arm (Fig. 2d).

### Family Soricidae

**Figure 3:**
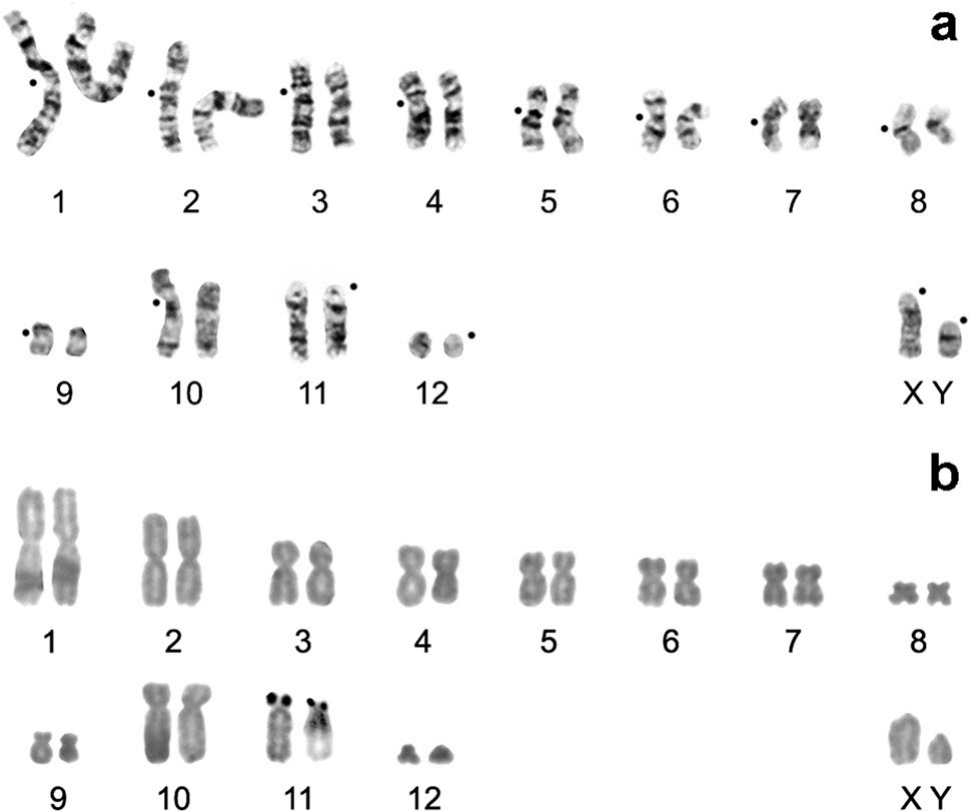
A male karyotype of *Sorex bedfordiae* from Sichuan Province (Songpan County, Xiaobaosi vil.): (**a**) G-banding, (**b**) silver nitrate staining. 2n = 26, NF*a* = 44. XY: sex chromosomes. Centromere positions are marked by black points.

As for lesser striped shrews (*Sorex bedfordiae* Thomas, 1911), nine specimens (G17-77, G17-81, G17-82, G17-83, G17-86, G17-89, G18-171, G18-172, G18-175 and G18-176) were collected at all sites except No. 3 and 4. The diploid chromosome number in all the karyotypes is 2n = 26, NF*a* = 44 (Fig. 3a). The autosomal set consists of eight large-to-small meta- or submetacentric pairs (chromosomes 1–8), one small and one large subtelocentric pair (chromosomes 9 and 10) and one large (chromosome 11) and one smallest (chromosome 12) acrocentric pair (Table 1). The X and Y chromosomes are represented by the mid-size and small acrocentrics, respectively. Silver nitrate staining revealed terminal localisation of nucleolus organiser regions (NORs) on short p-arms of the large acrocentric pair (chromosome 11) only (Fig. 3b).

**Figure 4:**
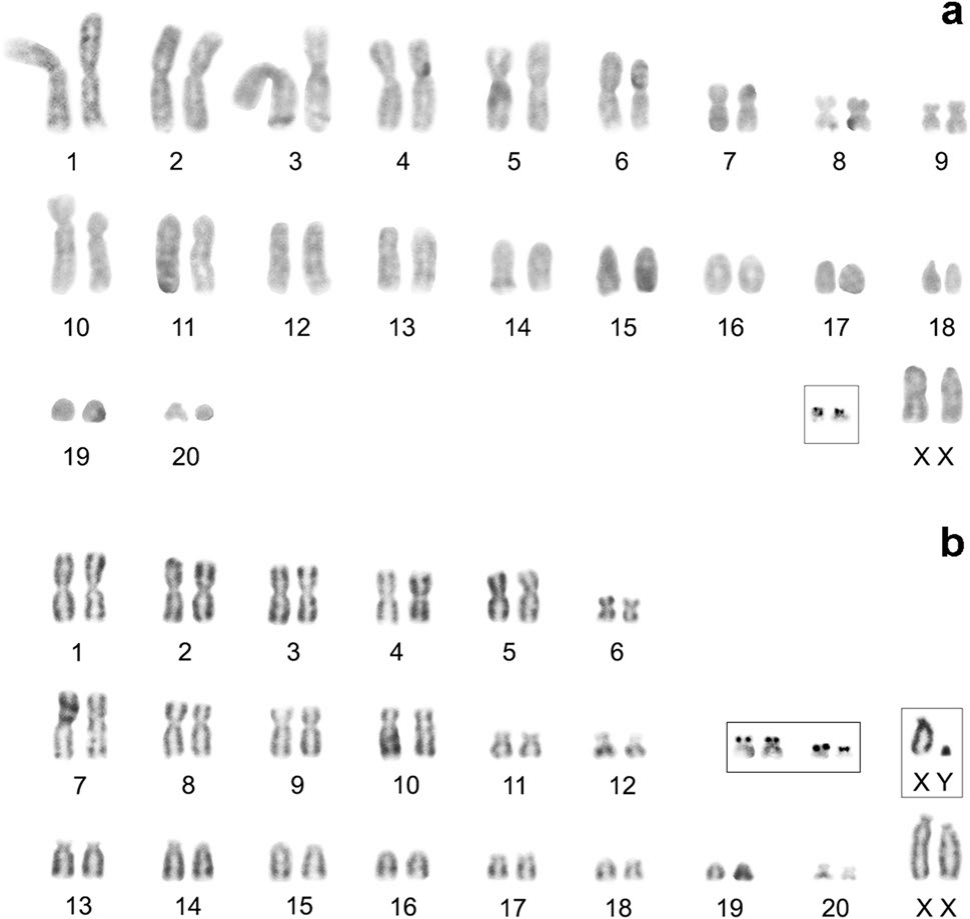
Conventionally stained karyotypes. (**a**) *Sorex thibetanus* from Gansu Province (Lianhuashan), 2n = 42, NF*a* = 60; (**b**) *Sorex cansulus* from Sichuan Province (Songpan County), 2n = 42, NF*a* = 64. XX: female sex chromosomes. Male sex chromosomes XY are boxed. Pairs bearing NORs in both karyotypes are boxed.

One female individual of the Tibetan shrew *S. thibetanus* Kastschenko, 1905 (G18-2), endemic to China, was collected at site No. 2. The karyotype is presented for the first time: 2n = 42, NF*a* = 60 (Fig. 4a). The autosomal set consists of nine large-to-small meta- or submetacentric pairs (chromosome 1–9), one large subtelocentric pair (chromosome 10) and 10 medium-to-small acrocentric pairs (chromosomes 11–20). Two mid-size acrocentrics were identified as X chromosomes. Silver nitrate staining revealed terminal localisation of NORs on short p-arms of a small acrocentric pair only (Fig. 4a, inset).

For the Gansu shrew *S. cansulus* Thomas, 1912, endemic to China, three specimens (G17-72, G18-79 and G18-80) were collected at sites No. 4 and 5. The karyotype is presented for the first time: 2n = 42, NF*a* = 64 (Fig. 4b). The autosomal set is composed of 20 bi-armed and eight single-armed autosomes. Among them, there are five large metacentric pairs (chromosomes 1–5) and one small metacentric pair (chromosome 6), four large (chromosomes 7–10) and two small (chromosomes 11 and 12) submetacentric pairs and eight medium-to-smallest subtelocentric pairs (chromosomes 13–20). Two large acrocentrics and a dot-like acrocentric are XX and Y chromosomes, respectively (Fig. 4b, inset). Silver nitrate staining revealed terminal localisation of NORs on short p-arms of two small bi-armed pairs (Fig. 4b, inset).

**Figure 5:**
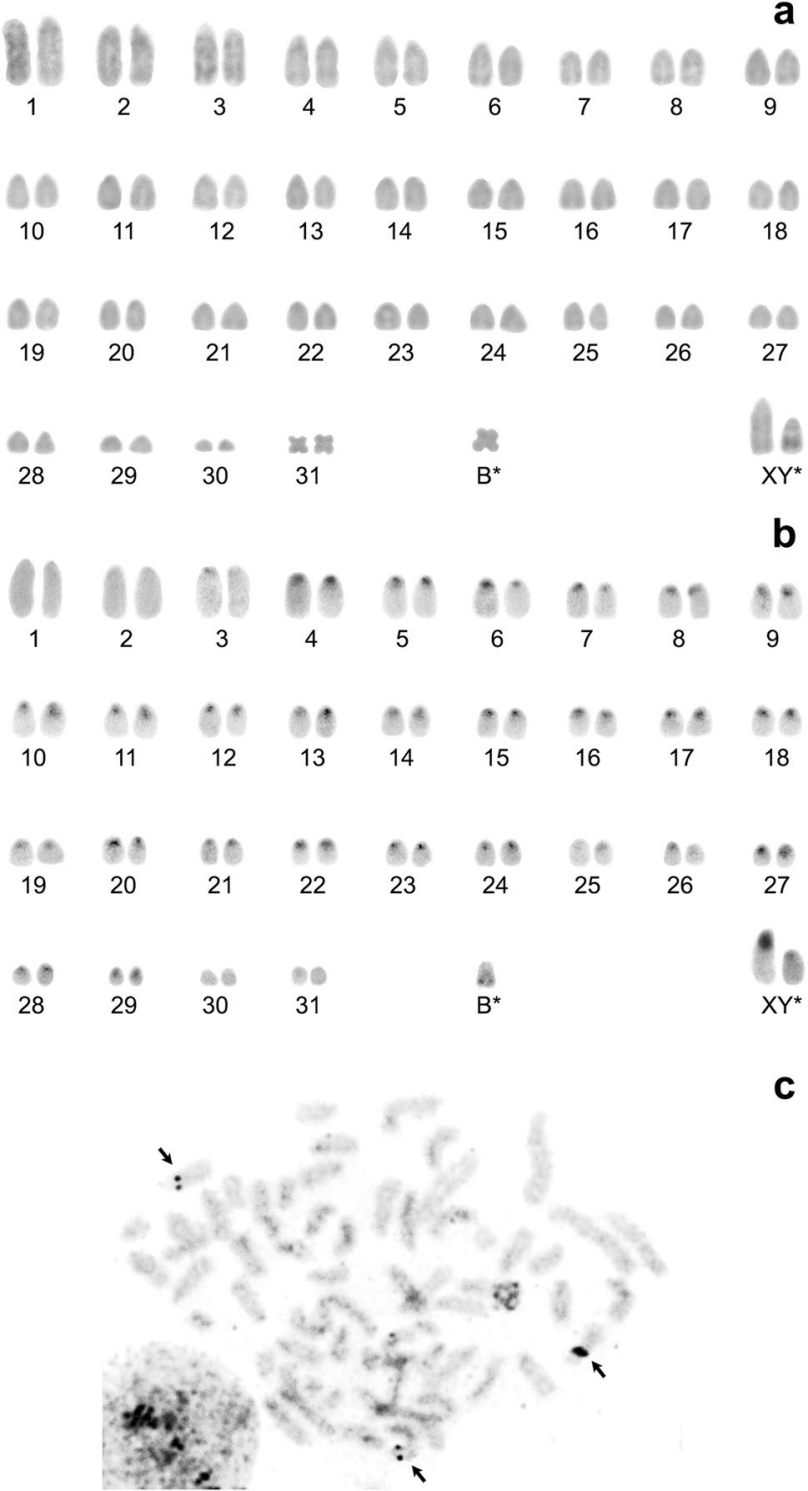
A male karyotype of *Chodsigoa hypsibia* from Gansu Province (Zhuoni County). (**a**) Conventional staining; (**b**) C-banding; 2n = 65, NF*a* = 66 (including a B chromosome); (**c**) silver staining. Black arrows indicate the locations of NORs. Asterisks indicate possible chromosome variants (see the main text for details). XY: sex chromosomes.

As for De Winton’s shrew *Ch. hypsibia* (de Winton, 1899), endemic to China, one male (G18-290) was collected at site No. 3. The karyotype is presented for the first time: 2n = 65, NF*a* = 66. The autosomal set consists of 30 large-to-small acrocentric pairs (chromosomes 1–30) gradually decreasing in size, one small metacentric pair (chromosome 31) and an unpaired small metacentric chromosome, which apparently is a B chromosome (Fig. 5a).

C-heterochromatic blocks were revealed in pericentromeric regions of 27 acrocentric pairs (chromosomes 3–29). Two large acrocentric pairs (chromosomes 1 and 2) and one smallest acrocentric pair (chromosome 30) as well as the small metacentric pair are C-negative. Slightly visible blocks of interstitial heterochromatin were noted on the B chromosome. Because one acrocentric chromosome has a large pericentromeric C-block, we identified it as an X chromosome (Fig. 5b). The Y chromosome was tentatively identified as an acrocentric chromosome and therefore is marked by an asterisk (as is the B chromosome). Silver nitrate staining uncovered centromeric localisation of NORs in three medium-to-small acrocentrics (Fig. 5c).

**Figure 6:**
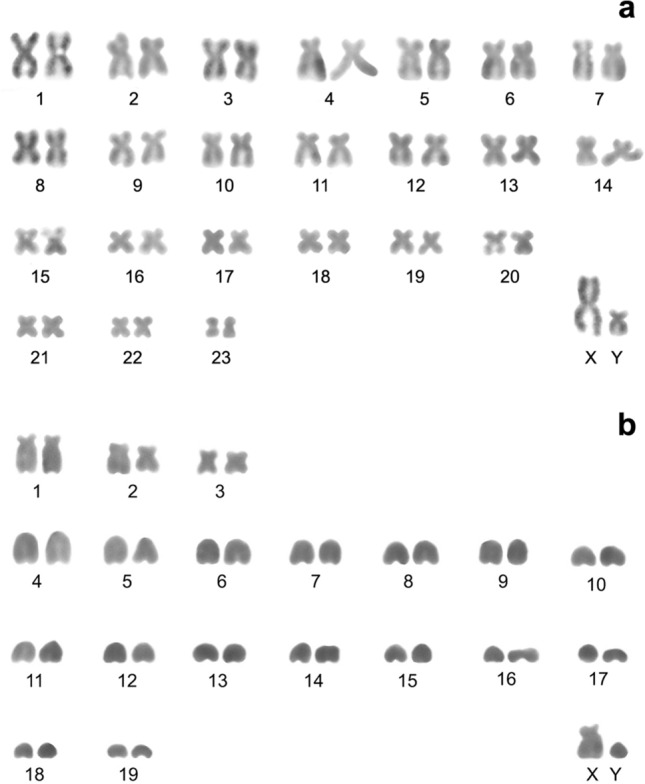
Conventionally stained male karyotypes. (**a**) *Anourosorex squamipes* from Gansu Province (Zhuoni), 2n = 48, NF*a* = 92; (**b**) *Crocidura suaveolens* from Sichuan Province (Zoige city), 2n = 40, NF*a* = 44. XY: sex chromosomes.

Two specimens of the Chinese mole shrew *A. squamipes* Milne-Edwards, 1872 (G17-140 and G18-336), were collected at sites No. 1 and 3. The studied male and female karyotypes do not differ from those reported previously^19^: 2n = 48, NF*a* = 92. The autosomal set is composed of 23 large-to-small meta- or submetacentric pairs gradually decreasing in size. In a male karyotype, the largest metacentric and small subtelocentric were identified as the X chromosome and Y chromosome, respectively (Fig. 6a).

A male individual of the lesser white-toothed shrew *C. suaveolens* Pallas, 1811 (G17-68), was caught at site No. 5. The diploid chromosome number was found to be 2n = 40, NF*a* = 44 (Fig. 6b). The autosomal set consists of three bi-armed pairs (chromosomes 1–3) and 16 single-armed pairs (chromosomes 4–19). The X chromosome is represented by a medium subtelocentric chromosome, and the Y chromosome by a small acrocentric.

## Discussion

Among the seven studied species of the order Eulipotyphla from southern Gansu and northern Sichuan Provinces, China, four species were analysed cytogenetically for the first time (*U. aff. soricipes*, *Ch. hypsibia*, *S. thibetanus* and *S. cansulus*).

In Talpidae, the most primitive subfamily Uropsilinae includes a single genus (*Uropsilus*), which formerly consisted of *Uropsilus andersoni*, *U. gracilis*, *U. soricipes*, *U. investigator* and *U. aequodonenia*^20,21^; later, *U. nivatus* and *U. atronates* were also recognised as valid species^9^. One more new species, *U. dabieshanensis* sp. nov., was recently described based on morphological and molecular genetic data^22^; thus, now the genus is thought to comprise seven species. At least 15 monophyletic lineages of *Uropsilus* have been recognised using molecular markers, and it seems that the taxonomic diversity of this genus may still be underestimated^9,22^. Until now, karyotypes of only two species, *U. nivatus* and *U. andersoni*, have been reported. Note that during the description of the karyotype^23^, *U. nivatus* was regarded as a subspecies of *U. gracilis*^24^ but was later elevated to full species rank^9^. The karyotype was described for an individual from an area inhabited only by *U. nivatus*; therefore, we use this name for the species, whose karyotype has been reported by Kawada et al.^23^. Despite the same 2n = 34, these karyotypes differ in chromosomal morphology as evidenced by a difference in NF*a*, which is 46 and 52 in *U. nivatus* and *U. andersoni,* respectively^13,23^. This variation is caused by a difference in the number of the subtelo- and acrocentric autosomes. Because only the conventional karyotype is reported for *U. andersoni*, it is difficult to compare the homologous arms and determine possible rearrangements between these two species. Moreover, the variation in the size of the short arms can be a consequence of some nuances of obtaining the chromosome preparations and then can lead to incorrect determination of NF*a*. Altogether, the authors^13^ concluded that the karyotype of *U. andersoni* is similar to that of *U. nivatus.*

The *U. aff. soricipes* karyotype (2n = 36, NF*a* = 56), described here for the first time, differs from those of the two species mentioned above by smaller numbers of meta- and submetacentric pairs (two pairs instead of four in both *U. nivatus* and *U. andersoni*) and a larger number of subtelo- and acrocentric pairs (in total, 15 pairs instead of 12). The comparison of homologous chromosome arms between the *U. nivatus* and *U. soricipes* karyotypes indicates that all chromosome arms match between the two species, judging by the G-band pattern. Two small acrocentric pairs (chromosomes 15 and 16) in *U. soricipes* correspond to metacentric pair No. 2 in the *U. nivatus* karyotype. Accordingly, the lower 2n in *U. nivatus*, and apparently in *U. andersoni*, can be explained by a single Robertsonian fusion resulting in a new metacentric pair. Moreover, these species show a difference in the size of the acrocentric Y chromosome, which is larger in *U. aff. soricipes* than in the other two species. The silver nitrate staining performed on *U. nivatus* and *U. soricipes* chromosomes did not reveal any differences between the karyotypes (the NOR is located within a secondary constriction in subtelocentric pair No. 3 in both species). Thus, more detailed cytogenetic data are needed to investigate karyotype evolution within the genus *Uropsilus.*

Here we present new findings about the karyotypes of six species of soricids, three of them are described karyotypically for the first time: *Ch. hypsibia*, *S. thibetanus* and *S. cansulus.*

*S. bedfordiae* belongs to the east Tibetan group of striped shrews, which includes *S. cylindricauda* and *S. excelsus*^7,25^. Previous cytogenetic research has uncovered intraspecific karyotypic variation within *S. bedfordiae*; 2n = 24–26 and 28 have been reported (Yunnan^26^; Sichuan^13^; southern Gansu^27^). In the present study, we found no polymorphic variants among the nine karyotyped individuals; all the shrews have equal chromosome sets: 2n = 26, NF = 46. In our previous work, karyotypes of two females from Lianhuashan Natural Reserve (site No. 2) were reported^27^ to contain a smaller number of chromosomal arms: 2n = 26, NF = 44. After re-checking of that material, it turned out that those two individuals are characterised by the same karyotype structure (2n = 26, NF = 46) as the three shrews described here.

Previously, karyotypes with 2n = 26, NF = 46 were detected in three *S. bedfordiae* individuals from Mt. Laojun (3900 m a.s.l.), Lijiang District in northern Yunnan province^26^. With the exception of one much larger subtelocentric pair (chromosome 10), those karyotypes were very similar to those described here. The lack of G-banded karyograms for the individuals from Yunnan makes it impossible to compare the homology of chromosomes between them in more detail. Additionally, these authors identified a 2n = 28 karyotype with an additional pair of small metacentrics. Nevertheless, they hypothesised that either it belongs to another species that closely resembles *S. bedfordiae*, e.g. *Sorex cylindricauda*, or the additional metacentric pair is the B chromosome.

**Figure 7:**
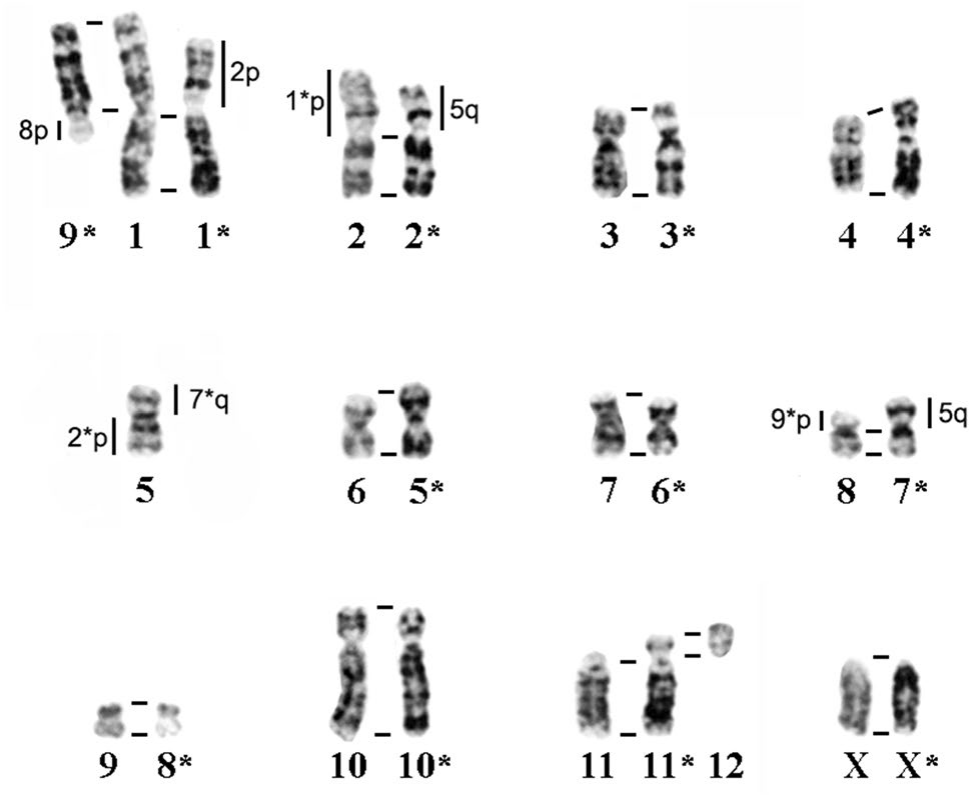
G-band matching between *Sorex bedfordiae* karyotypes. Homologous chromosomes of the 2n = 26 specimen presented in this paper (from Sichuan Province, Songpan County, Xiaobaosi vil.) are numbered in accordance with the Fig. 3, while those of a 2n = 24 specimen (taken with permission from Ref.^13^) are marked by an asterisk. Homologous regions are indicated by vertical and horizontal lines. XX: sex chromosomes.

Among the eight *S. bedfordiae* specimens from Mount Emei (3000 m a.s.l.) in central Sichuan Province^13^, two karyotype variants have been found: ‘2n = 24, NF = 46’ and ‘2n = 25, NF = 48’, which differ from each other by the presence of a supernumerary (B) metacentric chromosome in the latter. Besides a lack of B chromosomes, all the 2n = 26, NF = 46 karyotypes described in our study are characterised by the presence of an additional small acrocentric pair (chromosome 12) and a different length of one subtelocentric pair (chromosome 11, Figs. 3, 7).

A comparison of homologous chromosome arms between these G-banded karyotypes (2n = 24/25 and 2n = 26 from this study) indicated that they differ by several structural rearrangements (Fig. 7). Among the autosomes, a Robertsonian translocation (centric fusion) between telocentric pairs No. 11 and 12 in 2n = 26 karyotype resulted in the formation of a large subtelocentric pair No. 11* in 2n = 24/25 karyotype. Moreover, whole-arm translocations were detected in chromosome pairs Nos. 1, 2, 5, and 8 in 2n = 26 karyotype (Fig. 7). Nevertheless, all chromosome arms matched in the G-band pattern between the two *S. bedfordiae* karyotypes.

Molecular genetic studies point to the existence of eight highly divergent mitochondrial lineages among the shrews affiliated with the ‘cylindricauda’ group (five within *S. bedfordiae*, two in *S. excelsus* and one in *S. cylindricauda*) occurring on the eastern and southeastern edge of the QTP^7^. We did not aim to revise the intraspecific taxonomy of this group; however, it is worth noting that on the basis of the geographic origin and published genetic data^25^, all the karyotyped individuals presented here correspond to clade B, while those that have been reported by Moribe et al.^26^ and Motokawa et al.^13^ belong to other mitochondrial clades. Therefore, in this case, the molecular genetic differences match the karyotypic ones.

According to the recent phylogenetic analysis^25^, the Tibetan shrew belongs to the ‘*minutus* s.l.’ group together with *S. minutissimus*, *S. gracillimus* and a species endemic to Honshu Island, *S. hosonoi*. Nonetheless, karyotypic characteristics of *S. gracillimus* and *S. minutissimus* are close to those of the ‘caecutiens’ group but not those of the ‘*minutus* s.str.’ group^16,28^. Among these four species, *S. gracillimus* has a lower diploid number of chromosomes, 2n = 36, NF*a* = 60^29^, whereas both *S. hosonoi*^30^ and the *S. thibetanus* described here are characterised by the same number, 2n = 42, but differ in NF*a*: at 60 and 66, respectively. The karyotypic status of *S. minutissimus* is still not clear; 2n = 38 and 42 have been reported for the species from two distinct areas, Finland^31^ and Siberia^32^. Despite the absence of a 2n = 42 karyogram, in both cases, those authors reported^33^ that the karyotypes consist of mostly bi-armed autosomes and single-armed sex chromosomes with the same NF*a*, 72. It is worth noting that the *S. thibetanus* karyotype contains 10 bi-armed autosomes, which is the smallest number of these among the other three species. Except for our results on *S. thibetanus*, there are no data on the localisation of NORs in these species*.*

On the basis of molecular data, the Tibetan shrew has been estimated to have diverged from other members of ‘*minutus* s.l.’ (most likely from *S. gracillimus*) 2.0–2.5 million years ago (Mya)^25^. This age corresponds to the earliest possible time of QTP colonisation by the Tibetan shrew. The karyotypic data overall match the data from molecular genetic analysis indicating that *S. thibetanus* is affiliated with the eastern branch of the ‘*minutus* s.l.’ group^25^.

The latest multilocus nuclear and mitochondrial phylogeny^25^ of the nominal subgenus *Sorex* s.str. revealed that the ‘*caecutiens*’ species group is composed of five members: *S. caecutiens*, *S. isodon*, *S. unguiculatus*, *S. shinto* and *S. cansulus.* Among them, karyotypes of the first four species are known to be very similar (2n = 42, NF*a* = 64–66)^16,34,35^. The chromosomal set of the Gansu shrew *S. cansulus* has been unknown; here, for the first time, we demonstrated that this species has the same number of chromosomes, 2n = 42. A conventionally stained *S. cansulus* karyotype (NF*a* = 64) turned out to be quite similar in its morphological structure to species with NF*a* = 64 (*S. isodon*, *S. unguiculatus* and *S. shinto*). It has been documented that G-banding patterns are very conserved among *S. caecutiens*, *S. shinto* and *S. unguiculatus* karyotypes, which differ from each other by pericentric inversions^29^. The karyotype of *S. caecutiens* (NF*a* = 66) differs^35^ from these species by an additional pericentric inversion in pair No. 8 and a centromeric shift in pair No. 12. There are no data on differentially stained karyotypes of the other species.

In *S. cansulus*, NORs were detected for the first time in satellite regions of two small telocentric pairs, No. 19 and 20. The same localisation of NORs has been found in *S. unguiculatus*^29^. As Zima et al.^16^ have mentioned, ‘all species of this group with 42 chromosomes possess pronounced satellites in two small autosomal pairs’. Evidently, these autosomes bear NORs in all species, but only *S. cansulus* and *S. unguiculatus* karyotypes have been stained.

According to molecular data, the Gansu shrew separated from its most probable sister group (*S. caecutiens*) 1.5–2.0 Mya^25^, which is somewhat later in comparison with *S. thibetanus*. Consequently, one may theorise that the Gansu shrew is a more recent coloniser than the Tibetan shrew. Our karyotypic data overall match the data from molecular genetic analysis suggesting that *S. cansulus* belongs to the eastern branch of the ‘*caecutiens* s.l.’ group^25^.

Within the subfamily *Soricinae*, the tribe *Anourosoricini* is composed of four species^20^: *Anourosorex assamensis* Anderson, 1875, *A. schmidi* Petter, 1963, *A. squamipes* Milne-Edwards, 1872, and *A. yamashinai* Kuroda, 1935. Among them, karyotypic data have not been published for *A. schmidi*. *A. yamashinai*, endemic to Taiwan, has been studied using a set of differential stains and has 2n = 50, NF*a* = 96^36^, whereas only conventionally stained karyograms are available for *A. squamipes* (2n = 48, NF*a* = 92) collected in Fuleshan, Mianyang-shi, Sichuan Province, China^19^, and for *A. assamensis* from Tiddim Town of Chin State, western Myanmar (2n = 50, NF*a* = 96)^37^. Our results show that the structure of the *A. squamipes* routine karyotype does not differ from that reported previously: 2n = 48, NF*a* = 92. It has been demonstrated that the *A. yamashinai* karyotype^19^ contains two pairs of the large subtelocentrics that are missing in *A. squamipes.* Thus, species of the genus *Anourosorex* share a similarity of the karyotype, even though they are geographically distant from each other, as is the case, for example, for *A. yamashinai* (Taiwan) and *A. assamensis* (south-western China, south-eastern Tibet, north-eastern India and west-central Myanmar).

The taxonomic status of shrews of the genus *Chodsigoa* has changed many times. Previously, eight species have been recognised in the genus^5,20^, then new species, *Chodsigoa hoffmanni* sp. nov., was described on the basis of sequencing data from one mitochondrial and two nuclear genes^8^. Now nine species are attributed to the genus.

De Winton’s shrew *Ch. hypsibia* is endemic to China and occurs in central and southern provinces: south-eastern Qinghai, southern Gansu, southern Shaanxi, eastern Tibet, Sichuan, northern Yunnan and southern Anhui^2^. So far, karyotypic data on the genus *Chodsigoa* have been limited to one chromosome set that is described for a species endemic to Taiwan, *Chodsigoa sodalis* (2n = 44, NF = 88)^38^. The conventionally stained karyotype of this species consists of all bi-armed chromosomes; sex chromosomes have not been identified. The *Ch. hypsibia* karyotype stands out because all chromosomes are acrocentrics except one small metacentric pair of autosomes (chromosome 31) and an unpaired metacentric B chromosome (2n = 65, NF*a* = 66). On the basis of C-banding patterns, the X and Y sex chromosomes in the studied male were identified as a large acrocentric and medium acrocentric, respectively.

The large differences between the *Ch. hypsibia* (almost completely acrocentric) and *Ch. sodalis* (almost completely metacentric) karyotypes may be explained by the more ancient origin of *Ch. hypsibia*. To some extent, this notion is confirmed by large molecular genetic differences between *Ch. hypsibia* and most of other species of this genus. In the genus *Episoriculus*, closely related to *Chodsigoa*, there are also large differences in the number of metacentric and acrocentric chromosomes between *E. caudatus* with 2n = 60, NF = 118 (M + SM = 19, ST = 9, A = 1) and *E. soluensis* with 2n = 74, NF = 126 (M + SM = 12, ST = 13, A = 11)^39^. Those authors consider *E. soluensis* a subspecies of *E. sacratus soluensis*.

The lesser white-toothed shrew *C. suaveolens* inhabits a huge area, from Europe to Asia. The species is characterised by a mostly stable karyotype with 2n = 40, NF = 50, but some authors have reported chromosome sets 2n = 41–42, NF = 52, 54, which result from the presence of supernumerary (B) chromosomes (see review^16^). In a previous work, a 2n = 40, NF = 50 karyotype was identified in a female individual of *C. suaveolens* from the vicinity of the Goin Ba village in southern Gansu Province^27^. The specimen described here has 2n = 40 but a smaller number of chromosomal arms, NF = 48, owing to three bi-armed chromosome pairs instead of four. Unfortunately, highly condensed chromosomal arms in a low-quality chromosomal suspension did not allow us to clearly distinguish a possible fourth pair of bi-armed chromosomes. Thus, we cannot be completely sure that this specimen differs by a lower fundamental number of arms from those reported previously.

In a previous report, we showed that lesser white-toothed shrews from the QTP are close relatives of *Cr. suaveolens s.str.* (p-distance ~ 3%)^27^. Therefore, we can assume that this species is a relatively recent coloniser of the QTP and has not managed to achieve considerable genetic differentiation.

## Conclusion: the QTP is a natural laboratory of speciation

The eastern edge of the QTP features high species diversity of small mammals, including insectivores (Eulipotyphla); this phenomenon can be attributed to high habitat richness of this relatively small area. This effect is caused by the alternation of mountain ranges with well-defined high-altitude vegetation belts as well as by low mountains with elements of subtropical vegetation. On the tops of some ridges directly connected to the QTP, there are plateaus with steppe and tundra vegetation, while the high-altitude parts of other remote ridges are isolated and form the so-called sky island^27,40,41^.

On the one hand, the high species diversity is a product of multiple faunal exchange events between the QTP and more northerly areas. In our case, Tibetan and Gansu shrews as well as the lesser white-toothed shrew belong to phyletic lineages that have their centres of origin in Northern Eurasia (Siberia and Amur Oblast). At present, geographic ranges of Tibetan and Gansu shrews are separated from those of their closest sister groups by a gap of 1000–1500 km. It must be pointed out that colonisation from the north can hardly be a one-time event; the exact dispersal routes remain to be elucidated.

On the other hand, autochthonous taxa showed high inter- and intraspecific diversity in our study (*Chodsigoa*, *Uropsilus* and the *Sorex cylindricauda* species group), consistently with long-term persistence of these species at the eastern edge of the QTP resulting in the presence of forms at different stages of speciation. In contrast to autochthonous species, no intraspecific variation was detected among the recent colonisers. This also applies to the Tibetan shrew, whose separation from the ‘*minutus* s.l.’ group occurred more than 2 Mya. This observation suggests that autochthonous species have inhabited this area much longer.

The mountains at the eastern edge of the QTP can be called a natural laboratory of speciation, where both genetic and chromosomal alterations may promote the formation of new species. Further comprehensive comparative analyses of chromosomal and genetic variation within species groups of this region will enable an investigator to evaluate their contributions to barriers to gene flow. For instance, the genus *Anourosorex* features stable karyotypes and significantly lower genetic diversity than that in the other groups of species possessing similar geographic ranges, e.g. the ‘*cylindricauda*’ group. Nevertheless, this phenomenon may be explained alternatively as follows: representatives of the genus *Anourosorex* are more eurytopic, and the boundaries between habitats, including the ‘sky of islands’, are less important for them than for other groups. In any case, we propose that the high species diversity of the QTP derives from the combined effects of genetic and ecological processes.

## Materials and methods

### Compliance with ethical standards

The study was carried out in compliance with the ARRIVE guidelines. All applicable international, national and/or institutional guidelines for the Care and Use of Animals were followed. The collection of animals and the trapping method complied with the guidelines of the A.N. Severtsov IEE, RAS (Moscow), and the Institute of Zoology, CAS (Beijing). The experimental protocols were approved by the Bioethical Committee on Animal and Human Research of the A.N. Severtsov IEE, RAS (permission No. 30 issued on February 27, 2019), following all relevant guidelines and regulations. This article does not contain any experiments on human subjects performed by any of the coauthors.

### Specimen collection and identification

Animals were caught alive at seven sites in Gansu and Sichuan Provinces of China during a joint Russian–Chinese scientific expedition conducted as part of scientific project ‘RFBR–NSFC’ in September–October 2017–2018 using home-made live-traps^42^. These traps work well for assessing the species diversity of small mammals^43^. Capture locations were determined via a GPS (Garmin) personal navigation system (Fig. 1).

Regarding the Chinese shrew mole, on the basis of morphological characteristics and the geographic location, we tentatively assigned the found individual to *U. soricipes*^5^. Given that recent studies showed that the number of species in the genus *Uropsilus* may be much larger than is currently known^9,22^, we cannot be confident that this individual is a true *U. soricipes*. In this study, it is referred to as *U. aff. soricipes*. Further molecular genetic research is needed to clarify its true identity.

### Cytological preparations

Eighteen specimens belonging to seven species were karyotyped. Mitotic chromosome suspensions were made in the field from short-term culture of bone marrow and/or spleen after colchicine treatment in vivo as described previously^44^. For *U. aff. soricipes*, a chromosome suspension from primary fibroblast culture was prepared following the standard technique^45^. Air-dried chromosome spreads of all the specimens were conventionally stained with 2% Giemsa for 1–2 min and sequentially subjected to differential staining.

The standard trypsin/Giemsa staining procedure was carried out for the identification of each chromosome arm by G-bands^46^. C-banding was performed by the classic technique^47^. NORs were detected by silver nitrate staining^48^.

A Leica DFC-295 CCD camera mounted on a DM1000 (Leica) microscope or a Metasystems CCD (Zeiss) camera mounted on an AxioScope 2 (Zeiss) microscope was employed to capture images using MetaSystems Ikaros ver.5.3 and Leica Application ver.3.2 software packages, respectively.

## References

[1] Chen Z-Z (2021). Morphology and phylogeny of scalopine moles (Eulipotyphla: Talpidae: Scalopini) from the eastern Himalayas, with descriptions of a new genus and species. Zool. J. Linn. Soc..

[2] Burgin, C., He, K., Haslauer, R., Sheftel B., Jenkins, P., Ruedi, M., Hintsche, S., Motokawa, M., Hinckley, A., Hutterer, R. Family Soricidae (Shrews). In *Handbook of the Mammals of the World. Vol. 8. Insectivores, Sloths and Colugos* (eds. Wilson, D. E. & Mittermeier, R. A.) 395–551 (Lynx Edicions, Barcelona, 2018).

[3] Best, T. L. Family Erinaceidae (Hedgehogs and Gymnures). In *Handbook of the Mammals of the World. Vol. 8. Insectivores, Sloths and Colugos.* (eds. Wilson, D. E. & Mittermeier, R. A.) 288–330 (Lynx Edicions, Barcelona, 2018).

[4] Ai HS (2018). Taxonomic revision of the genus Mesechinus (Mammalia: Erinaceidae) with description of a new species. Zool. Res..

[5] Hoffmann, R. S. & Lunde, D. Order Soricomorpha. In *A Guide to the Mammals of China* (eds. Smith, A. T. & Xie, Y.) 297–327 (Princeton University Press, 2008).

[6] Kryštufek, B., Motokawa, M. Family Talpidae (Moles, Desmans, Star-nosed moles and Shrew moles). In *Handbook of the Mammals of the World. Vol. 8. Insectivores, Sloths and Colugos.* (eds. Wilson, D.E. & Mittermeier, R. A.) 552–619 (Lynx Edicions, Barcelona, 2018).

[7] Chen S (2015). Molecular phylogenetics and phylogeographic structure of Sorex bedfordiae based on mitochondrial and nuclear DNA sequences. Mol. Phylogenet. Evol..

[8] Chen ZZ (2017). Integrative systematic analyses of the genus Chodsigoa (Mammalia: Eulipotyphla: Soricidae), with descriptions of new species. Zool. J. Linn. Soc..

[9] Wan T (2018). Climate niche conservatism and complex topography illuminate the cryptic diversification of Asian shrew-like moles. J. Biogeogr..

[10] Lin W, Min ZL (1989). A study on karyotypes of two species of genus Hemiechinus (*H. dauuricus* and *H. hughi*). J. Northwest Univ..

[11] Pavlova SV (2018). First cytogenetic analysis of lesser gymnures (Mammalia, Galericidae, Hylomys) from Vietnam. Comp. Cytogenet..

[12] Zaitsev, M. V., Voyta, L. L., Sheftel, B. I. *The Mammals of Russia and adjacent territories. Lipotyphlans*. (2014).

[13] Motokawa M, Wu Y, Harada M (2009). Karyotypes of six soricomorph species from Emei shan, Sichuan province, China. Zool. Sci..

[14] Bulatova, N. S., Biltueva, L. S., Pavlova, S. V., Zhdanova, N. S., Zima, J. Chromosomal differentiation in the common shrew and related species. In *Shrews, Chromosomes and Speciation (Cambridge Studies in Morphology and Molecules: New Paradigms in Evolutionary Bio* (eds. Searle, J. B., Polly, P. D., Zima, J.) 134–184 (Cambridge University Press, 2019).

[15] Ye J (2006). Cross-species chromosome painting unveils cytogenetic signatures for the Eulipotyphla and evidence for the polyphyly of Insectivora. Chromosom. Res..

[16] Zima, J., Lukacova L., M. M. Chromosomal evolution in shrews. In *Evolution of shrews* (eds. Wojcik, J. M. & Wolsan, M.) 175–218 (Mammal Research Institute, Polish Academy of Science, 1998).

[17] Rieseberg LH (2001). Chromosomal rearrangements and speciation. Trends Ecol. Evol..

[18] Pavlova, S. V. & Searle, J. B. Chromosomes and speciation in mammals. In *Mammalian Evolution, Diversity and Systematics* (eds. Zachos, F. & Asher, R.) 17–38 (De Gruyter, 2018). 10.1515/9783110341553-002.

[19] Motokawa M, Harada M, Lin LK, Wu Y (2004). Geographic differences in karyotypes of the mole-shrew Anourosorex squamipes (Insectivore, Soricidae). Mamm. Biol..

[20] Hutterer, R. Order Soricomorpha. In *Mammal Species of the World: A Taxonomic and Geographic Reference, Volume 1* (ed. Wilson, D.E., Reeder, D. M.) 220–311 (The Johns Hopkins University Press, 2005).

[21] Wan T, He K, Jiang XL (2013). Multilocus phylogeny and cryptic diversity in Asian shrew-like moles (Uropsilus, Talpidae): Implications for taxonomy and conservation. BMC Evol. Biol..

[22] Hu T-L (2021). Description of a new species of the genus Uropsilus (Eulipotyphla: Talpidae: Uropsilinae) from the Dabie Mountains, Anhui, Eastern China. Zool. Res..

[23] Kawada S, Li S, Wang Y, Oda S (2006). Karyological study of Nasillus gracilis (Insectivora, Talpidae, Uropsilinae). Mamm. Biol..

[24] Hoffmann RS (1984). A review of the Shrew-moles (Genus Uropsilus) of China and Burma. J. Mammal. Soc. Jpn..

[25] Bannikova AA (2018). Evolutionary history of the genus Sorex (Soricidae, Eulipotyphla) as inferred from multigene data. Zool. Scr..

[26] Moribe J, Li S, Wang Y, Kobayashi S, Oda S (2009). Sorex bedfordiae has the smallest diploid chromosomme number of the XY Group in the Genus Sorex (Mammalia, Soricidae). Cytologia (Tokyo)..

[27] Sheftel BI (2018). Notes on the fauna, systematics, and ecology of small mammals in Southern Gansu, China. Biol. Bull..

[28] Ivanitzkaya E, Kozlovsky AI, Orlov VN, Kovalskaya YM, Baskevich MI (1986). New data on karyotypes of common shrews (Sorex, Soricidae, Insectivora) in fauna of the USSR. Zool. Zhurnal.

[29] Tada T, Obara Y (1988). Karyological relationships among four and subspecies of sorex revealed differential staining techniques. J. Mamm. Soc. Jpn..

[30] Moribe J, Noro T, Kobayashi S, Oda SI (2007). The karyotype of the Azumi shrew Sorex hosonoi. Acta Theriol. (Warsz).

[31] Halkka O, Skaren U, Halkka L (1970). The karyotypes of Sorex isodon Turov and *S. minutissimus* Zimm. Ann. Acad. Sci. Fenn. Ser. A IV Biol..

[32] Orlov VN, Kozlovsky AI (1971). A synopsis of chromosome complements of shrews of the genus Sorex. Vestn. Mosk. Univ. Biol. i Počvovedenie.

[33] Zima J, Kral B (1984). Karyotypes of European mammals. 1. Acta Sci. Nat. Brn.

[34] Fedyk S, Ivanitskaya EY (1972). Chromosomes of Siberian shrews. Acta Theriol. (Warsz).

[35] Biltueva LS (2000). Comparative chromosome analysis in three Sorex species: *S. raddei*, *S. minutus* and *S. caecutiens*. Acta Theriol..

[36] Harada M, Takada S (1985). Karyotypes of two species of insectivora from Taiwan (Insectivora, Soricidae). Experientia.

[37] Kawada S, Kurihara N, Tominaga N, Endo H (2014). The first record of Anourosorex (Insectivora, Soricidae) from Western Myanmar, with special reference to identification and Karyological characters. Bull. Natl. Museum Nat. Sci. Ser. A.

[38] Motokawa M, Harada M, Lin LK, Cheng HC, Koyasu K (1998). Karyological differentiation between two Soriculus (Insectivora: Soricidae) from Taiwan. Mammalia.

[39] Motokawa M, Harada M, Mekada K, Shrestha KC (2008). Karyotypes of three shrew species (*Soriculus nigrescens*, *Episoriculus caudatus* and *Episoriculus sacratus*) from Nepal. Integr. Zool..

[40] Headl WF (1951). Sky islands of Arizona. Nat. Hist..

[41] He K, Jiang X (2014). Sky islands of southwest China. I: An overview of phylogeographic patterns. Chin. Sci. Bull..

[42] Shchipanov NA (1986). On the ecology of lesser white-toothed shrew (*Crocidura suaveolens*). Zool. Zhurnal.

[43] Shchipanov NA, Litvinov YN, Sheftel BI (2008). Rapid method for estimating local biodiversity of a community of small mammals. Contemp. Probl. Ecol..

[44] Pavlova SV, Borisov SA, Timoshenko AF, Sheftel BI (2017). ‘European’ race-specific metacentrics in East Siberian common shrews (Sorex araneus): A description of two new chromosomal races, Irkutsk and Zima. Comp. Cytogenet..

[45] Freshney RI (2010). Culture of Animal Cells: A Manual of Basic Technique and Specialized Applications.

[46] Seabright M (1971). A rapid banding technique for human chromosomese. Lancet.

[47] Sumner AT (1972). A simple technique for demonstrating centromeric heterochromatin. Exp. Cell Res..

[48] Howell WM, Black DA (1980). Controlled silver-staining of nucleolus organizer regions with a protective colloidal developer: A 1-step method. Experientia.

